# Effects of Ectoine on Behavior and Candidate Genes Expression in ICV-STZ Rat Model of Sporadic Alzheimer’s Disease

**DOI:** 10.15171/apb.2017.075

**Published:** 2017-12-05

**Authors:** Niloofar Bazazzadegan, Marzieh Dehghan Shasaltaneh, Kioomars Saliminejad, Koorosh Kamali, Mehdi Banan, Reza Nazari, Gholam Hossein Riazi, Hamid Reza Khorram Khorshid

**Affiliations:** ^1^Genetics Research Center, University of Social Welfare and Rehabilitation Sciences, Tehran, Iran.; ^2^Laboratory of Neuro-organic Chemistry, Institute of Biochemistry and Biophysics (IBB), University of Tehran, Tehran, Iran.; ^3^Reproductive Biotechnology Research Center, Avicenna Research Institute, ACECR, Tehran, Iran.

**Keywords:** Sporadic Alzheimer disease, Ectoine, Gene expression, Morris Water Maze test, STZ- rat model

## Abstract

***Purpose:*** Alzheimer’s disease (AD) is pathologically defined by the presence of amyloid plaques and tangles in the brain, therefore, any drug or compound with potential effect on lowering amyloid plaques, could be noticed for AD management especially in the primary phases of the disease. Ectoine constitutes a group of small molecule chaperones (SMCs). SMCs inhibit proteins and other changeable macromolecular structures misfolding from environmental stresses. Ectoine has been reported successfully prohibit insulin amyloid formation in vitro.

***Methods:*** We selected eight genes, DAXX, NFκβ, VEGF, PSEN1, MTAP2, SYP, MAPK3 and TNFα genes which had previously showed significant differential expression in Alzheimer human brain and STZ- rat model. We considered the neuroprotective efficacy by comparing the expression of candidate genes levels in the hippocampus of rat model of Sopradic Alzheimer’s disease (SAD), using qPCR in compound-treated and control groups as well as therapeutic effects at learning and memory levels by using Morris Water Maze (MWM) test.

***Results:*** Our results showed significant down-regulation of Syp, Mapk3 and Tnfα and up-regulation of Vegf in rat’s hippocampus after treatment with ectoine comparing to the STZ-induced group. In MWM, there was no significant change in swimming distance and time for finding the hidden platform in treated comparing to STZ-induced group. In addition, it wasn’t seen significant change in compound-treated comparing to STZ-induced and control groups in memory level.

***Conclusion:*** It seems this compound may have significant effect on expression level of some AD- related genes but not on clinical levels.

## Introduction

Alzheimer’s disease (AD) is the most prevalent type of dementia among aged people which is clinically bolded by continuous memory loss and a slow deterioration in cognitive function.^1^ AD is neuropathologically specified by loss of neurons and synapses, especially in the hippocampus and cortex, the extracellular agglomeration of neuritic plaques, containing amyloid-β (Aβ) peptide, and the presence of intracellular neurofibrillary tangles (NFT) composed of hyperphosphorylated tau protein.^2-6^ The great majority of AD cases are sporadic with aging, type 2 diabetes and apolipoprotein E4 as the essential risk factors.^7^ Other mechanisms may commence before the emergence of tau and Aβ pathologies in sporadic AD pathogenesis.^8,9^ These mechanisms include vascular pathology,^10^ mitochondrial dysfunction,^11^ oxidative stress,^12^ hypoxia,^13^ insulin resistance,^14^ and chronic neuroinflammation.^15^

A nomination causal event in sporadic Alzheimer’s disease (SAD) is distracted brain insulin metabolism.^16^ Early abnormalities in brain glucose/energy metabolism are pronounced in parietotemporal and frontal areas with high glucose requirement and high insulin sensitivity which suggests damaged insulin signaling in the pathogenesis of SAD.^17,18,19^ Injecting streptozotocin, a glucosaminenitrosourea toxic to pancreatic β cells, into rat brain induced the phosphorylation of the tau protein, amyloid deposits and other SAD symptoms.^19-21^ The aims of management in AD patients have been to ameliorate or at least slow the loss of memory and cognition and to preserve independent function. Acetylcholinesterase suppressors are first-line factors for the remedy of mild to moderate AD.^22-25^ Even though small changes in action mechanisms, these inhibitors have different detrimental effects.^26,27^ The most accepted adverse effects are nausea, vomiting, and diarrhea; cardiovascular and neurological adverse effects are comparable. The incidence of negative effects is precisely related to the dose administered.^27^ Therapy with rivastigmine, donepezil or galantamine as Acetylcholinesterase inhibitors for six months to one year resulted in kind of enhanced cognitive function.^23^ Improvements in daily behavior and activities also were eminent in patients cured with one of these factors; however, none of them has a major therapy efficacy and the clinical importance of these effects is ambiguous. Whenever there is no effective treatment for AD, substances with efficient inhibition of the amyloid formation have been sought as drug candidates for AD management.^28-31^

Ectoine is a heterocyclic amino acid or a partially hydrogenated pyrimidine derivative (1,4,5,6-tetrahydro-2-methyl-4-pyrimidinecarboxylic acid.^32^ Ectoines are common solutes of aerobic heterotrophic bacteria and compose a class of small molecule chaperones (SMCs). SMCs stack to high intracellular concentrations, inhibiting the misfolding of proteins and other unstable macromolecular structures from environmental stresses.^33-36^ SMCs have already known as eminently effective in maintaining enzymatic activities against heating, freezing and drying.^33,37^ Regarding to a reporting, SMCs like ectoine, betaine, trehalose, citrulline could successfully constrain insulin amyloid formation *in vitro* (Arora, Ha et al. 2004).^38^ All findings propose the effect of SMCs against amyloid formation, which may make them permanent drug candidates for medicating neurodegenerative diseases in the future. Ectoine maintains proteins and enzymes from proteolysis, thermal stress and change of the pH or the salt concentration. Studies showed its potency to maintain various proteins, nucleic acids, membranes and whole cells.^33,37,39-42^ Many genes have been reported to be related with AD which they have shown expression changes in Alzheimer model and human brain comparing to normal group.^43,44^ Among them *DAXX*, *NFκβ*, *VEGF* genes with the role in apoptosis, inflammation and angiogenesis showed significant statistical diversity in Alzheimer human brain.^43^ Furthermore, *Psen1*, *Mtap2*, *Syp*, *Mapk3* and *Tnf α* genes with the role in γ-secretase, cytoskeleton, synapses, kinase and inflammation showed significant statistical diversity in STZ rat model.^44^

Regarding possible mechanisms of AD like inflammation and oxidative stresses in the brain, and role of various genes in these proceedings, the neuroprotective efficacy was investigated by comparing the expression levels of the mentioned genes in the hippocampus of rat model of SAD using qPCR in treated and untreated groups. Moreover, the remedial effects were considered at learning and memory levels as well.

## Materials and Methods

**Figure 1:**
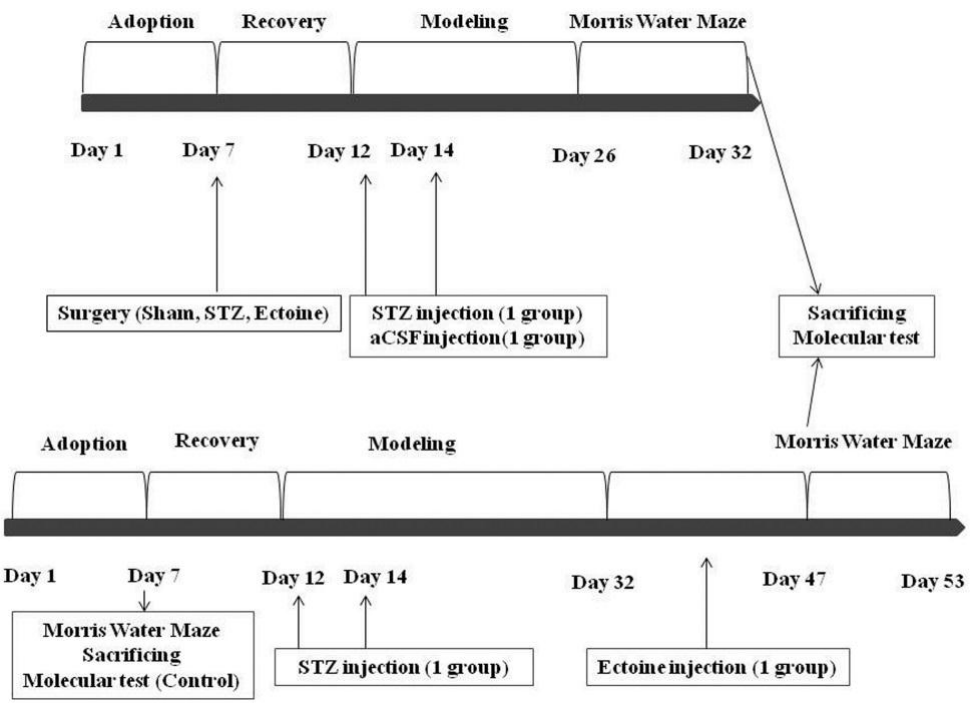
The schematic figure is representative of all procedures. Four rat groups were grouped as control, sham, STZ and Ectoine-treated after adoption days and recruited in treatments and tests

Thirty one mature male *Wistar* rats with 250-300 g weight were used in this research. They were kept in cage with adequate food and water, in a stable environment at 22°C and 12h light/dark cycle.^45^ Animals were distributed into four groups each containing of seven to eight rats: the control group (Eight rats) received no medication and surgery; the sham group (Eight rats) had bilateral intracerebroventricular (ICV) injection of aCSF as the vehicle of STZ; the Alzheimer group (Seven rats) which received bilateral ICV infusion of STZ, and the treated STZ group (Eight rats) which received the compound for fifteen days after modeling as intrapritoneal injection (6 mg/day).^38^ All groups except the control one had five recovery days after surgery and before treatment with aCSF, STZ and compound- treated. All procedures were shown in details in Figure 1.

Learning and memory examined for all group of rats using Morris Water Maze (MWM) test after treatment.^46^ The maze was a round black pool with 150 cm diameter, 60 cm height. Maze was filled with water to a depth of 40 cm with temperature of about 23°C. Four identical spaced places at the circumference of the pool divided the pool into four quadrants, and were used as beginning parts. An escape platform of 10 cm diameter was located 2 cm beneath the surface of the water at a fixed position in the center of one of the quadrants. All rats were participated to a daily session of four training trials for five consecutive days. Each rat was permitted to find the latent platform within sixty seconds. The time spent to detect the platform (Escape latency), the distance each rat swam to find the platform (Path Length) and the swimming speed (Velocity) were recorded. One day after acquisition, a probe test was performed to evaluate memory level by removing platform.^47^

After MWM test, they were immolated and all hippocampi were dissected and reserved in RNA protector solution at -20°C.^48^All processes were performed according to the National Institute of Health Guide for the care and use of laboratory animals.^49^

Total RNAs were extracted from hippocampus tissues using UP100H ultrasonic processor (Germany) and RNeasy Plus Mini Kit (Qiagen, Hilden, Germany) conferring to the manufacturer's protocol. Purity and integrity of RNAs were specified using Nano-drop spectrophotometer and gel electrophoresis. cDNA synthesis was performed using RevertAid^TM^ First Strand cDNA Synthesis Kit (Fermentas, Thermo Fisher Scientific) according to the manufacturer's protocol.

The relative expression levels of the eight genes *Daxx, Nfkb, Vegf, Psen1*, *Mtap2*, *Syp*, *Mapk3* and *Tnf α* in rat hippocampus of each group were detected using SYBR green Real Time PCR (Takara SYBR Master Mix (Shiga, Japan) in ABI 7500 Real-time PCR system (Applied Biosystem, Foster city, CA, USA) and). All genes expression normalizations were done by *Actb* endogenous control.^50,51^ Cycle threshold (Ct) values were used to calculate fold changes in gene expression between groups using REST 2009 software. P-values less than 0.0125 for analysis by REST and in other analysis less than 0.05 were considered statistically significant. MWM test data were analyzed by GraphPad Prism 6 software; Kruskal Wallis (Dunn’s multiple comparisons test) test was used for three recorded factors (escape latency, path length and the swimming speed) in all treated and untreated groups separately during five days.

## Results

### MWM Test Results

**Figure 2:**
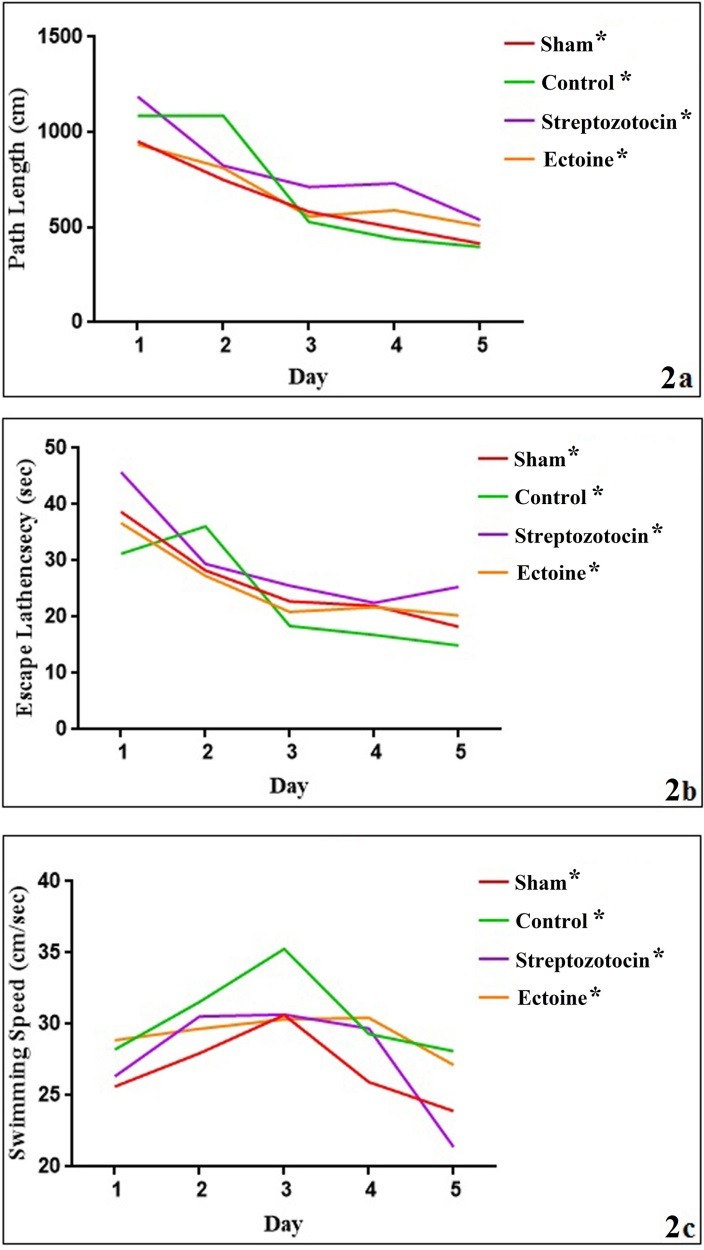
The mean value of path length (swimming distance), escape latency (time for finding hidden platform) and swimming speed (velocity) during five continuous days in all treated and control groups were represented in 1a, 1b and 1c respectively. (*) is representative of significant difference in swimming distance and time for finding hidden platform throughout trial days in all groups. Kruskal Wallis (Dunn’s multiple comparisons test) test was used for three recorded factors (escape latency, path length and the swimming speed) in all treated and untreated groups separately during five days

**Figure 3:**
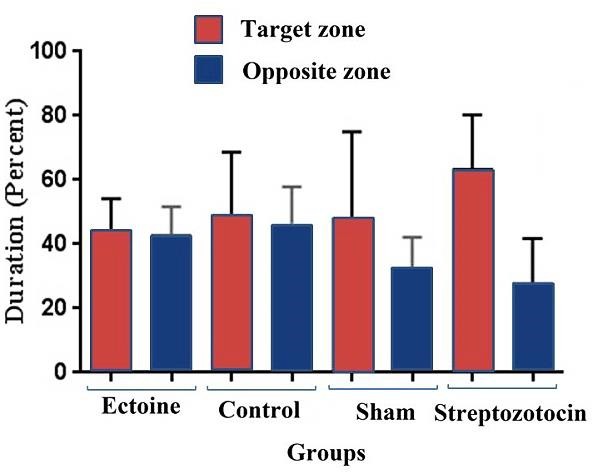
Inability of rats to find the hidden platform was assessed by probe test. The median with interquartile range of percentage of time spending in target (The zone in which hidden platform located) and opposite zone (The opposite zone of target zone) in the sixth day (four trials) of the test in all treated and control groups. Data were analyzed by GraphPad Prism 6 software

After evaluating the learning and memory level changes using MWM test, our results represented a remarkable gradual decrease in swimming distance and time for finding the hidden platform during five days in all groups, despite of no significant change in these two criteria in -treated comparing to the STZ-induced group during five days, Figure 2a, b and c are representative of MWM test analyses among treated and untreated groups. The swimming speed did not show significant change during five trial days among groups except in STZ group. Probe test results showed no significant memory change in - treated comparing to STZ-induced and control groups (Figure 3).

### Expression of Candidate Genes

**Figure 4:**
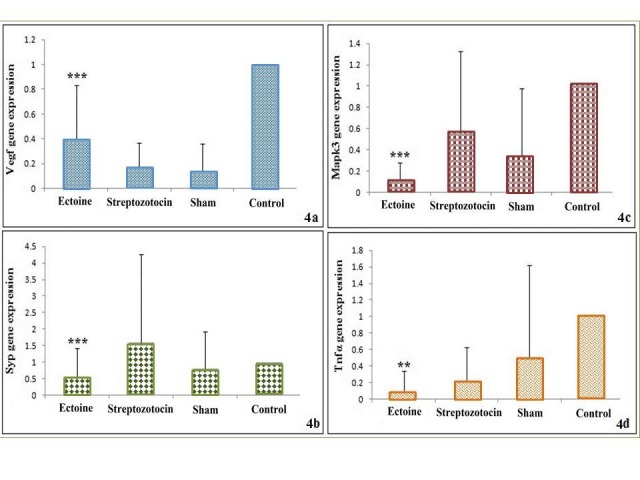
The expression level of *Vegf, Syp, Mapk3* and *Tnf-α* genes in all treated and control groups are shown in this figure. Significant expression changes of Ectoine-treated group comparing to Alzheimer group were labeled out with asterisk

*Syp, Mapk3* and *Tnf α* genes were down-regulated and* Vegf* gene was upregulated in rat’s hippocampus after two weeks treatment with comparing to STZ-induced group (Table 1). Statistically significant change (p-value ≤ 0.01) was observed in expression level of *Syp, Mapk3, Tnf α* and* Vegf* with decreasing ~ 3, 4.5 and 2- fold in the *Syp, Mapk3* and* Tnf α* also increasing 2- fold in *Vegf* (Figure 4). As it is obvious in Figure 4b,* Syp* showed no significant up-regulation in the STZ-induced comparing to the control group also, in the -treated comparing to the STZ- induced group there was a significant down-regulation (P-value= 0.002). Also in Figure 4c no significant down-regulation of *Mapk3* gene was observed in the STZ-induced comparing to the control group, whereas significant 4.5-fold down-regulation was seen in the -treated comparing to the STZ-induced group (P-value=0.000). As it is obvious in Figure 4d, *Tnfα* showed significant down-regulation in STZ-induced comparing to control group (P-value= 0.008) the same in -treated versus STZ-induced group with about 2- fold reduction (P-value= 0.01). Figure 4a is representative of the expression of *Vegf which* was remarkably decreased in the STZ-induced comparing with the control group (P-value= 0), but its expression showed about 2-fold significant up-regulation in the -treated comparing to the STZ-induced group (P-value= 0.001).

**Table 1:** The table is representative of the eight gene expression levels in Ectoine-treated comparing to the Alzheimer (STZ) group. Asterisk shows significant p-value

**Gene**	**Ectoine-treated/STZ**	**p-value**	**Up or down regulation**
*Daxx*	1.9	0.117	Up-regulation
*Nfκb*	0.7	0.288	Down- regulation
*Vegf*	2.2	0.001*	Up-regulation
*Syp*	0.36	0.002*	Down-regulation
*Psen1*	1.05	0.823	Up-regulation
*Mapk3*	0.2	0.000*	Down-regulation
*Mtap2*	0.6	0.095	Down-regulation
*Tnfα*	0.45	0.017*	Down-regulation

## Discussion

In this study, we evaluated expression of eight candidate genes (*Daxx, Nfkb, Vegf Psen1*, *Mtap2*, *Syp*, *Mapk3* and *Tnf α*) for Alzheimr’s disease in RNA level in AD rat model. Our results showed that three genes, *Syp, Mapk3* and *Tnf α* were significantly down-regulated in the treated compared to the STZ-induced group as same as up-regulation of* Vegf*.

Synaptic loss and dysfunction are reported to be the molecular basis of cognitive defect in AD.^52,53^ Chen et al. (2012) reported their finding as notably reduced expression of synaptophysin (*Syp*) in the hippocampus of icv-STZ mice. Also other Synapse-related genes were found not to be significantly reduced in STZ- induced model even some of them were up-regulated.^44^ In the present study, as it is obvious in Figure 4b,* Syp* showed no significant up-regulation in the STZ-induced comparing to the control group also, in the -treated comparing to the STZ- induced group there was a significant down-regulation.

Mitogen-activated protein kinase 3 (*Mapk3*) is an anti- apoptosis gene which is also involved in neuron plasticity .^44^ Protein phosphorylation regulates neuronal plasticity, APP processing and tau aggregation.^54^ Regarding studies, several protein kinases disorder in AD brain or play many roles in the disease. In the study by chen et al. (2012), gene expressions of some AD-related protein kinases showed down-regulation in STZ-induced mice, but only *Mapk3* expression change was significant. In our study no significant down-regulation of this gene was observed in the STZ-induced comparing to the control group, whereas significant 4.5-fold down-regulation was seen in the -treated comparing to the STZ-induced group.

Many evidences indicate that neuroinflammation can act as an independent factor at very early stage of AD, where the immune-related genes and cytokines are the important factors. It was reported that proinflammatory cytokines such as TNFα are elevated in the CSF and plasma of AD patients.^55^ Several biologic medications against TNFα have decreased Aβ deposition, behavioral impairments and inflammation in AD animal models,^56-59^ which suggest that TNFα is a deleterious agent in AD term and can serve as a reliable AD target. It has been reported that persistent neuronal TNFα expression in 3xTg AD mice led to large amount of neuronal loss.^60^ In present study, *Tnfα* showed significant down-regulation in STZ-induced comparing to control group the same in -treated versus STZ-induced group with about 2- fold reduction.

*VEGF* levels in AD patient have been controversial. According to the available data, it is postulated that reduced *VEGF* expression might be proved in AD.^61^ Increased levels of this gene in the hippocampal cortex of AD patients comparing to normal brain were reported.^62^ Some genetic studies have described that *VEGF* levels cause neurodegeneration in part by harming neural tissue perfusion.^63^ Gene array analysis has shown up-regulation of angiogenesis relevant genes in the AD brain.^64^ In our study, the expression of *Vegf* was significantly decreased in the STZ-induced compared to the control group, but its expression showed about 2-fold significant up-regulation in the -treated comparing to the STZ-induced group.

MWM test results represented a significant gradual decrease in swimming distance and time for finding the hidden platform during five training days in all groups, but not significant change was seen in the -treated comparing to the STZ-induced group. Probe test results showed no significant memory change in the compound-treated comparing to the STZ-induced and control groups.

According to the previous studies that were reported in the treatment of mild to moderate atopic dermatitis also, ectoine ameliorates ischemia reperfusion injury after intestinal transplantation in rats. Moreover, it causes recovery of neutrophil apoptosis in lung inflammation. Therefore, it can be effective systemically in different diseases.^65-67^ Regarding previous report, it could successfully prohibit insulin amyloid formation *in vitro.*^38^

## Conclusion

Finally, regarding to both behavioral and gene expression results, it could be concluded that ectoine may have significant effects on expression level of genes related to angiogenesis, synapsis, kinases and inflammation but, not on clinical level. It is recommended to use transgenic animals that could be very helpful to study gene expression levels in Alzheimer’s disease. Using transgenic animals could be used to study the effects of our compound in prevention of amyloid beta protein and fibrillary tangles formation. Moreover, using different doses of the ectoine maybe have more effective results on Alzheimer disease. Another study that could be helpful is to use the ectoine before construction of Alzheimer models to observe its effects in prevention of the disease. Duration of ectoine administration is another important factor which might be more efficient on Alzheimer treatment and at last route of administration, for instance intravenous (IV) instead of intraperitoneal could be resulted in better management of Alzheimer’s disease.

## Acknowledgments

We would like to thank all colleagues specially Dr. Saeed Talebi who helped us in gene expression analysis and Dr. Parvaneh Daneshmand for useful comments. The study was supported by the University of Social Welfare and Rehabilitation Sciences, Tehran, Iran.

## Ethical Issues

Not applicable.

## Conflict of Interest

Authors declare no conflict of interest in this study.
